# Computational methods using genome-wide association studies to predict radiotherapy complications and to identify correlative molecular processes

**DOI:** 10.1038/srep43381

**Published:** 2017-02-24

**Authors:** Jung Hun Oh, Sarah Kerns, Harry Ostrer, Simon N. Powell, Barry Rosenstein, Joseph O. Deasy

**Affiliations:** 1 Department of Medical Physics, Memorial Sloan Kettering Cancer Center, New York, NY 10065, USA; 2 Department of Radiation Oncology, University of Rochester Medical Center, Rochester, NY 14620, USA; 3 Department of Pathology, Albert Einstein College of Medicine, New York, NY 10461, USA; 4 Department of Radiation Oncology, Memorial Sloan Kettering Cancer Center, New York, NY 10065, USA; 5 Department of Radiation Oncology, Mount Sinai School of Medicine, New York, NY 10029, USA

## Abstract

The biological cause of clinically observed variability of normal tissue damage following radiotherapy is poorly understood. We hypothesized that machine/statistical learning methods using single nucleotide polymorphism (SNP)-based genome-wide association studies (GWAS) would identify groups of patients of differing complication risk, and furthermore could be used to identify key biological sources of variability. We developed a novel learning algorithm, called *pre-conditioned random forest regression (PRFR)*, to construct polygenic risk models using hundreds of SNPs, thereby capturing genomic features that confer small differential risk. Predictive models were trained and validated on a cohort of 368 prostate cancer patients for two post-radiotherapy clinical endpoints: late rectal bleeding and erectile dysfunction. The proposed method results in better predictive performance compared with existing computational methods. Gene ontology enrichment analysis and protein-protein interaction network analysis are used to identify key biological processes and proteins that were plausible based on other published studies. In conclusion, we confirm that novel machine learning methods can produce large predictive models (hundreds of SNPs), yielding clinically useful risk stratification models, as well as identifying important underlying biological processes in the radiation damage and tissue repair process. The methods are generally applicable to GWAS data and are not specific to radiotherapy endpoints.

Approximately 50% of cancer patients receive radiation therapy (RT) as part of their treatment at some stage during a course of treatment, resulting in a large number of survivors who are susceptible to the development of radiation-induced toxicities^1^,^2^,^3^. Despite advances in radiotherapy and biotechnology, some surrounding normal tissue is inevitably irradiated during a course of RT, which may lead to the development of side effects and a worsening quality of life for patients. A long-term goal of research in the field of radiation oncology has been to identify patients predisposed to severe complications who should therefore receive less dose or, potentially, other treatment modalities^4^. Non-sensitive patients could then be treated safely with effective doses.

It has been established that more than a hundred different genes are involved, to some extent, in the determination of radiosensitivity^5^. Efforts to find genetic markers that predict radiation response (called “radiogenomics”) have focused on identifying one or a small number of germ-line single-nucleotide polymorphisms (SNPs) with genome-wide statistical significance^6^,^7^,^8^,^9^. There has recently been substantial progress in identifying SNPs that are thought to be associated with radiation-induced toxicities in several cancers using increasingly large (genome-wide) data sets^4^. However, such single-SNP methods are limited with respect to their ability to identify SNPs that are significantly associated with an endpoint because of the need for massive multiple-testing corrections to limit false positive observations^4^,^10^, thereby limiting detection to relatively large effect sizes. For typical study sizes, such methods are unable to reliably identify modest effects from the many genes that are likely to contribute to the overall risk for polygenic processes such as tissue repair following radiation damage.

As an alternative approach, we hypothesize that predictive models with a large number of genetic markers (SNPs) are much more likely to be successful, because they allow for inclusion of small effects that individually do not reach genome-wide significance. Although some (even many) SNPs included in the resulting model are “false positives”, the final model merely needs to result in a reliable overall estimation of risk.

A key requirement for this approach is to design a computationally feasible, unbiased, and reliable algorithm that begins with the genome-wide scale of millions of SNPs^11^,^12^,^13^,^14^. This appears to be an attractive application for machine learning, which often focuses on building predictive models—even when the number of features is much larger than the number of training examples^15^. It is often said that a key drawback to machine learning is the “black box” nature of the algorithms. To the contrary, as we demonstrate, a machine learning-based ranking of the impact of a large number of genetic markers is a nearly ideal bioinformatics input to identify key underlying biology.

To address this problem, we designed a novel computational method that incorporates machine learning and bioinformatics techniques to predict individual radiosensitivity and to identify relevant biological processes. We tested our approach on a GWAS dataset, with the goal of predicting the risk of developing rectal bleeding and erectile dysfunction in prostate cancer patients treated with RT.

The American Cancer Society expects that 180,890 new prostate cancer cases will be diagnosed in 2016 with an estimated 26,120 deaths^16^. Radiotherapy for prostate cancer patients sometimes results in severe gastrointestinal (GI) toxicity such as rectal bleeding, ulcerations, or less commonly, strictures or fistulas^17^. Radiation induced-erectile dysfunction (ED) is also a common side effect, as well as other genitourinary (GU) severe toxicities such as dysuria, hematuria, and bladder ulceration or necrosis^18^,^19^. Accurately predicting who is at high or low risk of such complications has been elusive^20^. Hence, if successful, the method could have a significant impact on the ability to customize radiotherapy treatments for many patients, resulting in fewer severe treatment complications.

## Materials and Methods

### Data

We analyzed a GWAS cohort dataset, previously reported in refs 19 and 21, consisting of 368 prostate cancer patients treated with RT at a single institution. This study received Institutional Review Board approval at Mount Sinai Medical Center and all patients provided informed consent. All experimental protocols and procedures were performed in accordance with the guidelines of the Mount Sinai Medical Center. We included all patients with at least one year of followup after RT, for two late radiation-induced toxicities: rectal bleeding and erectile dysfunction. Rectal bleeding was graded using the discrete Radiation Therapy Oncology Group (RTOG) late radiation morbidity scoring schema. Grade 2 or higher requires medical treatment (see Appendix in ref. 21 for detailed information). Erectile dysfunction was assessed using the patient-administered Sexual Health Inventory for Men (SHIM) questionnaire (see ref. 22 for detailed information) with the following 5 grades: no erectile dysfunction (SHIM total score, 22−25), mild (17−21), mild to moderate (12−16), moderate (8−11), and severe (1−7). Patients with severe erectile dysfunction before RT were excluded from the analysis, i.e., patients with SHIM ≤ 7 or the Mount Sinai Erectile Function (MSEF) score ≥2 for those who were treated before introduction of the SHIM questionnaire. The MSEF scoring system is defined as follows: 0 = no erections; 1 = erections but insufficient for intercourse; 2 = erections suboptimal but sufficient for intercourse; and 3 = optimal erections^19^. For each endpoint, we defined cases and controls as follows: for rectal bleeding, cases and controls were defined as patients with the RTOG grade ≥2 and grade ≤1, respectively; and for erectile dysfunction, patients with at least 1 post-treatment SHIM ≤7 were considered cases and patients with all post-treatment SHIM ≥16 were considered controls, which was previously used in ref. 19. These definitions resulted in 236 evaluable patients for erectile dysfunction and 365 evaluable patients for rectal bleeding. For an unbiased assessment of resulting predictive models, the dataset was split at the outset, once and for all, into two groups: a training dataset (2/3 of samples) and a validation dataset (1/3 of samples). The split was random except we continued random selection until the event rate was nearly equal in the two groups. Table 1 shows the number of samples used in the training and validation datasets for each endpoint. Under the assumption of an additive effect genetic model, SNPs were coded with the number of copies of the minor allele^23^.

### Quality Control and Population Stratification

A quality control test for the GWAS data was performed, which excluded SNPs that did not fulfill the following criteria from the analysis: (1) missing rate >5%; (2) minor allele frequency (MAF) <5%; and (3) Hardy-Weinberg Equilibrium (HWE) p-value < 1 × 10^−5^, resulting in 606,571 SNPs remaining.

The measured genetic inflation factor was <1 and 1.06 for rectal bleeding and erectile dysfunction, respectively, indicating negligible evidence of population stratification. In addition, a chi-square test showed that there was no significant difference in the number of events (toxicities) between populations for both endpoints with p-value = 0.37 and 0.43 for rectal bleeding and erectile dysfunction, respectively (Supplemental Material).

### Model Building

Paul *et al*. proposed a statistical model building method called *pre-conditioning* consisting of supervised principal component analysis (SPCA) followed by least absolute shrinkage and selection operator (LASSO) for a regression problem with a continuous outcome variable^24^. The motivation is to replace the measured original outcome variable with another continuous outcome variable that has high correlation both with important features as well as the measured outcome variable, thus providing a more informative input for further statistical learning. We incorporate this concept by proposing a predictive model that we term *pre-conditioned random forest regression (PRFR)* wherein we first convert a binary outcome variable (toxicity vs. non-toxicity) to a continuous outcome variable using principal components and logistic regression, and thereafter build a predictive model using random forest regression. The modeling tree nature of the algorithm, and the ability to effectively use many SNPs as biomarkers across hundreds of trees, makes it an attractive machine learning method to apply to SNP GWAS data. Random forests have previously been employed to effectively model the genetic risk to heart disease^25^, and Parkinson’s disease and Alzheimer’s disease^26^.

Before the model building process, to remove irrelevant SNPs and to make the process computationally tractable, SNPs with univariate p-values > 0.001 are filtered out, based on a chi-square test with a 3 × 2 contingency table that consists of the counts of each genotype (i.e., common/common, common/rare, and rare/rare) vs. outcome (toxicity, no toxicity). Note that single-SNP association tests are conducted using only training data.

Model building steps are repeated using 5-fold cross-validation (CV) on the training data, repeated 100 times with random shuffling of samples. For each shuffling of the training data, the process is as follows: (1) individual SNPs are then ranked based on the resulting area under the receiver operating characteristic curves (AUCs) resulting from univariate logistic regression over 5-fold CV samples, (2) using an increasing number of the top ranked SNPs, principal component analysis (PCA) is applied, (3) the first two principal components are weighted within a multiple logistic regression model fitted to the outcomes. This results in continuous pseudo-outcomes (the “pre-conditioned outcomes”) that can also be viewed as preliminary estimates of complication probability, (4) the pre-conditioned outcomes used in the model building process are found in a way that the resulting AUC values reach saturation (around 1.00) from step (3), and (5) a random forest regression model is then constructed using all SNPs that passed the threshold of p-value 0.001. Model performance and variance are estimated by tabulating model performance on the hold-out validation dataset for each CV. Finally, a resulting predictive model built using the entire training dataset is assessed on the hold-out validation dataset by computing an AUC and examining a calibration plot. Algorithm S1 describes the proposed method.

Random forest regression is a well-known ensemble method, consisting of a collection of regression trees. Each tree sub-classifies each patient according to a subset of features that define the branches of the tree. Each tree is constructed using a bootstrap dataset that is randomly sampled with replacement from the original patient data, having the same size as the original data; likewise, a random subset of features is used at each node split. Trees are built by finding a best feature to create a branch at each level of the tree. The final answer is found by averaging over many trees (a “forest”), thus capturing fitting to detailed characteristics while being insensitive to the prediction bias of any single tree^14^,^26^. Variability in model performance was estimated on the hold-out validation data by random forest models built repeating the modeling building process (steps 1–5) 500 times (5-fold CV × 100 iterations) on the training data. Each random forest model consisted of 1000 trees.

At each node of each tree, a best SNP was chosen from a subset of SNPs (the size equals to the square root of the number of SNPs that passed the univariate threshold with a p-value of 0.001) randomly selected. The minimum number of samples required to populate a node was set to 5. With this threshold, the tree stops growing when the number of samples arriving at the terminal nodes is smaller than 5.

To better characterize this approach, we compared performance with several other approaches, using LASSO instead of random forest, but still with the pre-conditioned outcomes (denoted PL); using a standard random forest classifier with the original outcomes (denoted RFC); and using LASSO with the original outcomes (LLR).

### Identification of Enriched Biological Processes Associated with Endpoints

We investigated the possible relationship between genes tagged by important SNPs used in the model building process and biological processes implicated in the endpoints. To do this, we created an estimate of importance for SNPs used in the random forest model building phase. We randomly permutated SNPs in the random forest predictive model and ranked the impact on predictive accuracy for out-of-bag (OOB) data (i.e., samples not selected at the time of tree construction). It is reasonable to assume that SNPs with larger importance scores have a greater chance of being causally related to the endpoint than those with smaller importance scores. We selected the top 25% of SNPs ranked using the importance score, and further selected only those SNPs that fall within 10 kb upstream or downstream of known genes, as determined using the UCSC genome browser. We then used this filtered list of genes as input to a curated bioinformatics database (MetaCore, Thomson Reuters) that can generally identify significant biological processes and key gene/protein interactions given a list of input genes. This process was repeated with the top 50%, 75%, and 100% of SNPs that passed the univariate threshold. We chose a best cutoff to GO enrichment analysis that resulted in the most reasonable biological relevance^5^,^27^.

### Generating SNP Importance Scores

In more detail, we evaluated the importance of each SNP in the predictive random forest based on the following steps: (1) for each SNP, the prediction mean squared error (MSE) is computed in each tree using OOB data, (2) one SNP per tree in the OOB data is randomly permutated, whereas the remaining SNPs are left unchanged and the resulting MSE, denoted MSEp, is tabulated. MSEp is typically larger than MSE, and (3) these steps are iterated for all SNPs over the entire model building process. The difference between MSEp and MSE is averaged across all the trees and the averaged score is used as a final measure of individual SNP importance.

### Data availability

The GWAS data and clinical variables have been deposited in the dbGaP database (accession number: phs000772.v1.p1).

## Results

### Univariate Analysis using Training Data

**Figure 1:**
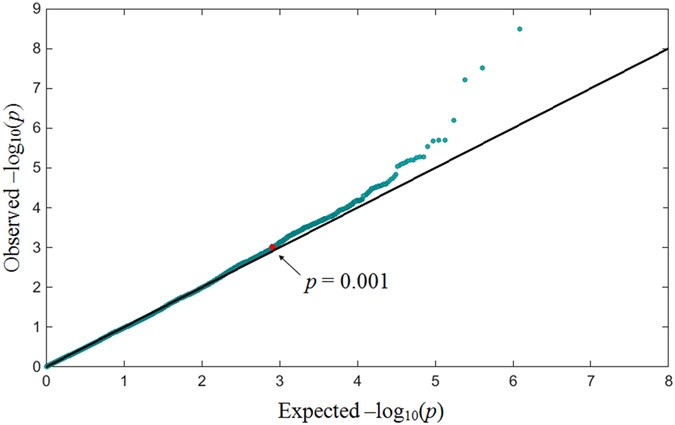
Quantile-Quantile (Q-Q) plot for p-values obtained by single association tests using chi-square test on the training dataset in rectal bleeding. It is typical of GWAS that many of the highest correlations are false-positives. We address this problem via multi-SNP machine learning methods.

Figure 1 shows a quantile-quantile (Q-Q) plot for rectal bleeding. Deviation from straight line behavior, as observed, supports the concept that a number of SNPs are involved with late radiation-induced rectal bleeding. Note that SNPs are not completely independent statistical predictors; hence in the x-axis p-values are only nominal. We filtered out SNPs with p-values > 0.001 computed in the single-SNP chi-square test on the training data, resulting in 749 and 367 SNPs that met the threshold for rectal bleeding and erectile dysfunction, respectively.

### Performance Evaluation of Predictive Models

**Figure 2:**
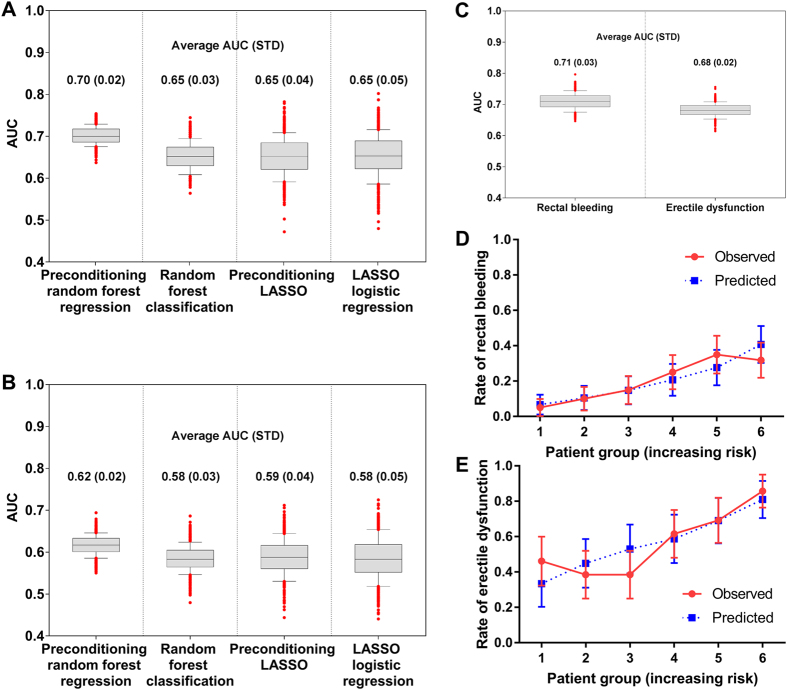
Performance evaluation of the proposed method on a validation dataset. Performance comparison of our proposed method (pre-conditioned random forest regression) with other computational methods on the hold-out validation data in (**A**) rectal bleeding and (**B**) erectile dysfunction. STD: standard deviation. (**C**) Box plots showing the performance of our proposed predictive model for rectal bleeding and erectile dysfunction, resulting from the list of SNPs obtained after the biological relevance test of gene ontology biological processes. For the erectile dysfunction model, two clinical variables (ADT and age) were also added in the model building process. The filled circle dot indicates an AUC when a final model for each endpoint, built using all training data, was tested on the hold-out validation data. For the final models, comparison of the predicted and actual incidence for (**D**) rectal bleeding and (**E**) erectile dysfunction on the hold-out validation data. The patients were sorted based on the predicted outcomes and binned into 6 groups with 1 being the lowest risk group and 6 being the highest risk group. The error bar indicates the standard error.

Figures 2A and B show box plots of the 500 AUC values (5-fold CV × 100 iterations), indicating predictive power of the computational methods for rectal bleeding and erectile dysfunction on the hold-out validation data tested by predictive models built using cross validation-based training data. For both endpoints, PRFR outperformed other methods, with average cross validation AUCs of 0.70 and 0.62 for rectal bleeding and erectile dysfunction, respectively, when the first two principal components were used in the PCA pre-conditioning phase (For rectal bleeding, using only the first principal component resulted in an average cross validation AUC of 0.69). There was no further performance improvement when the first 3, 4, and 5 principal components were used. In contrast, for erectile dysfunction, the predictive performance was saturated with the first principal component. Nonetheless, for consistency, we used the first two principal components for all modeling. The predictive performance of the three alternative methods was similar, but PRFR had the highest performance and smallest standard deviation (STD) on cross validation. The random forest-based models including PRFR and RFC had smaller STDs compared with the LASSO-based models. All final comparisons with the hold-out validation data and biological analyses were based on the PRFR model.

### Identification of Key Biological Processes Associated with Endpoints

**Figure 3:**
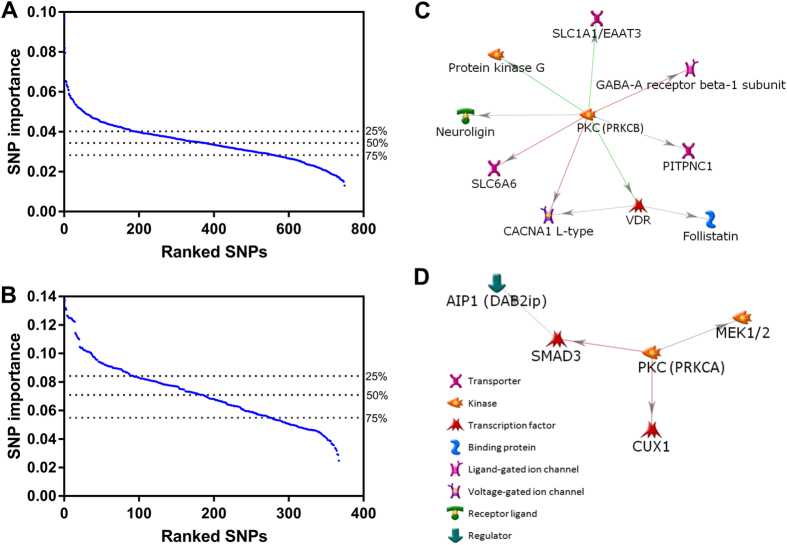
SNP importance score measured in the model building process for (**A**) rectal bleeding and (**B**) erectile dysfunction. The red dashed lines indicate the points of the top 25, 50, and 75% of SNPs. Directly connected protein-protein interaction networks for (**C**) rectal bleeding and (**D**) erectile dysfunction generated using the MetaCore software with the top 50% of SNPs. The line colors indicate the activation (green), inhibition (red), and unspecified (grey) effects.

For the 749 and 367 SNPs obtained in univariate analysis on the training data, associated with rectal bleeding and erectile dysfunction, respectively, we assessed the extent of importance of each SNP in the PRFR model. Figures 3A and B show the sorted importance score of these SNPs. The y-axis scale is an estimate of the mean square increased by permutation for each SNP on OOB samples. Using the top 25%, 50%, 75%, and 100% of these SNPs, we searched for genes within 10 kb upstream or downstream. With this threshold, 198 and 90 unique genes were found, respectively, for rectal bleeding and erectile dysfunction. Interestingly, there were no common SNPs between the two sets of SNPs, whereas 11 common genes were found, including: ANKS1B, CNTN4, DLG2, DPP6, ETV6, FHIT, GABRB1, MCTP2, PDE4D, PDZRN3, and TBC1D9. We uploaded each list of genes identified with a different percentage of the top SNPs into the curated MetaCore database, and performed GO enrichment analysis in order to identify associated biological processes for these genes^28^,^29^,^30^. For rectal bleeding, we found that the list of genes (133 genes) identified with the top 50% of SNPs (374 SNPs) was more biologically plausible than the list resulting from the use of 100% of SNPs. This should be expected, as the list of all SNPs relies only on the univariate threshold, whereas the list of the top 50% of SNPs relies on correctness of the modeling process, which cannot be dismissed given the good predictive accuracy of the final model. For erectile dysfunction, when the top 50% of SNPs (183 SNPs) were used, reasonable biological processes were also identified with 60 genes. Tables 2 and 3 show the top 10 biological processes implicated in either rectal bleeding or erectile dysfunction, along with the corresponding genes for each biological process. For all 10 biological processes in both tables, the estimated false discovery rate (FDR) was less than 0.05. Interestingly, CACNA1D and CXCR5 involve all 10 biological processes implicated in rectal bleeding and erectile dysfunction, respectively. Tables S1 and S2 give a complete list of the biological processes identified for the different percentage of the top SNPs.

With the expectation that interacting proteins in a biological network have a similar function, we also explored directly connected protein-protein interaction networks produced using the given genes. The largest connected networks consisted of 10 and 5 proteins for rectal bleeding and erectile dysfunction, respectively, and are shown in Fig. 3C and D.

Under the assumption that the univariate cutoff is a process prone to error, and that the importance scores are a more reliable indication of SNP importance, we rebuilt predictive models using the top 50% of SNPs (374 and 183 SNPs for rectal bleeding and erectile dysfunction, respectively) for both endpoints. The predictive power on the hold-out validation data for late rectal bleeding was (only) slightly improved with an average cross validation AUC of 0.71, whereas for erectile dysfunction there was greater improvement with an average cross validation AUC of 0.65.

### Improvement of Predictive Power by Adding Clinical Variables

We also investigated whether adding clinical variables could improve predictive power. Clinical variables include age, Gleason score, tumor volume, smoking, diabetes, hypertension, prostate-specific antigen (PSA), radiation dose computed as biologically equivalent dose (BED), and the use of androgen deprivation therapy (ADT). Among all 236 evaluable patients used in the erectile dysfunction analysis, 85 (85/133; 63.9%) and 35 (35/103; 34.0%) patients received ADT in the case and control groups, respectively; there were 52 (39.1%) and 36 (34.9%) smokers; 6 (4.5%) and 3 (2.9%) patients had diabetes; 48 (36.1%) and 32 (31.1%) patients had hypertension; the mean age in years was 65.7 (STD: 7.0) and 60.3 (STD: 6.4) in the case and control groups, respectively. In a logistic regression significance test for these clinical variables on the training data, no statistically significant variable was found for rectal bleeding, whereas for erectile dysfunction, age (p-value = 1.30 × 10^−5^; odds ratio = 1.14), ADT (p-value = 0.0002; odds ratio = 3.45), and Gleason score (p-value = 0.008; odds ratio = 1.89) were statistically significant. Smoking, diabetes, and hypertension were not statistically significant with p-values of 0.378, 0.410, and 0.522, respectively. Using the same threshold of p-value > 0.001 as used in filtering SNPs, the predictive model for erectile dysfunction was rebuilt by combining the two clinical variables (age and ADT) and the top 183 SNPs. As a result, predictive performance was further improved with an average AUC of 0.68 on the hold-out validation data after repeating 5-fold CV 100 times as shown in Fig. 2C.

### Results of the Final Model

The final PRFR model built using all the training data was tested on the hold-out validation data, and the predicted outcomes were compared with the observed outcomes. Patients were grouped into 6 bins of increasing risk, with 1 being the lowest risk group and 6 being the highest risk group. Figure 2D and E compare the predicted incidence of endpoints and the actual incidence of endpoints in each group. The resulting AUCs were 0.70 and 0.69 for rectal bleeding and erectile dysfunction, respectively. Good agreement between the predicted incidence and the actual incidence was seen, with chi-square test p-values of 0.95 and 0.93 for rectal bleeding and erectile dysfunction, respectively, indicating that the observations are statistically consistent with the model. As shown, the models are well calibrated, and track the validation data well.

## Discussion

Individual variation of normal tissue radiosensitivity depends on the combined influence of radiation dose, clinical variables, comorbidities, and genetic differences among patients^4^. This effort, called “radiogenomics”, has the ambitious goal of unravelling the association between genetic variants and clinical normal tissue radiosensitivity. To date, this has enabled identification of several SNPs associated with normal tissue injury in cancer patients treated with RT^7^,^8^,^31^. However, radiogenomics studies to date have relied on the classical single marker association test, which misses SNPs that make small, but real contributions to risk.

In our study, to predict the risk of individual radiosensitivity, we proposed a novel multi-SNP predictive model based on machine learning techniques. A hypothesis behind our approach is that although the impact of individual SNP on an endpoint is small, combining contributions of highly significant SNPs and those that are relatively weakly significant into a robust model can improve the capability of the model in predicting the risk of the endpoint. This apparently works well because (a) SNP GWAS are low-noise sources, compared to, say, mRNA arrays, and (b) damage of radiation repair is a highly polygenic process. Using a GWAS dataset, we demonstrated that our method outperformed other existing computational methods in predicting the risk of rectal bleeding and erectile dysfunction that are common radiation-induced toxicities in prostate cancer patients treated with RT.

We had previously shown that, for late rectal bleeding, the LASSO was a useful modeling method for the second step of our pre-conditioning algorithm. However, here we show superior results using random forests^4^. We also go beyond that report by analyzing the endpoint of erectile dysfunction and by demonstrating a method to connect SNP importance in the machine learning results to key biological functions.

Using the pre-conditioning idea, we converted the original binary outcomes (toxicity vs. non-toxicity) to continuous outcomes (pre-conditioned outcomes) that were used in the modeling (see Supplementary Figure S1). Our model with the pre-conditioned outcomes achieved much better predictive performance compared with computational methods with the original binary outcomes. Note that we used the pre-conditioned outcomes only for modeling purpose. The performance test was carried out in comparison between the predicted outcomes and the original binary outcomes for all models shown in Fig. 2. More specifically, two models including pre-conditioning random forest regression and pre-conditioning lasso used the pre-conditioned outcomes for modeling, whereas other two models including random forest regression classification and lasso logistic regression used the original binary outcomes for modeling. For all four models, the predicted outcomes on the validation data were compared with the original binary outcomes to evaluate predictive power. The pre-conditioned outcomes were produced using logistic regression with principal components (effectively, latent variables) such that the pre-conditioned outcomes are highly correlated with the original binary outcomes as well as a set of important SNPs, thereby denosing the original binary outcomes. The motivation for this approach is that the combination of two different ways of predicting complication probabilities would be likely to result in a more accurate model, assuming both have some validity and are not redundant. Pre-conditioning in some sense can be viewed as just a convenient method to combine prediction models.

To assess the variability of predictive performance in the proposed model, we used 5-fold CV. In addition, we tested our method using 60% of the validation samples that were randomly selected^32^. After 100 iterations, average AUC values were 0.70 and 0.64 for rectal bleeding and erectile dysfunction, respectively. That is, predictive power for rectal bleeding was the same as the method used in this study, whereas there was slight improvement for erectile dysfunction compared to AUC = 0.62 in this study. This test further supports the reliability of our proposed method.

As can be seen in Fig. 1, most SNPs are not associated with the endpoint. Removal of these irrelevant SNPs from further analysis greatly reduces computational cost. To that end, researchers have typically used a cutoff of p-values obtained in univariate analysis. However, it is challenging to find an optimal cutoff due to the tradeoff between eliminating true positive SNPs vs. keeping too many false positive SNPs. In this study, we initially employed a p-value threshold of 0.001, which has also been used in other studies for initial filtering^33^,^34^.

To probe the number of false positive SNPs included in the prediction model, we injected phoney SNPs into the rectal bleeding modeling process. To do this, we generated 60,000 synthetic SNPs using the GWAsimulator (http://biostat.mc.vanderbilt.edu/wiki/Main/GWAsimulator) relying on HapMap CEU data. With a p-value cutoff of 0.001, 74 SNPs remained. We combined these SNPs with 749 SNPs that were originally chosen in univariate analysis for rectal bleeding and iterated the model building process with the combined 823 SNPs (see Supplementary Figure S2). There were no noisy SNPs within the top 26 SNPs, suggesting that the most highly ranked SNPs contain many, though probably not all, real biomarkers. On the other hand, the inclusion of phoney SNPs in the model indicates that the model clearly balances true effects with statistical artifacts. The correlation with validation data outcomes demonstrates that a valid estimation of risk has been obtained.

We hypothesized that the predictive power of the models can be further improved by removing false-positive SNPs in the modeling and they can be detected by exploring biological processes via GO enrichment analysis. Using the SNP importance score measured in random forest regression, we identified nearby genes for the top 25%, 50%, 75%, and 100% of SNPs and performed GO enrichment analysis to investigate biological processes for these genes (Supplementary Table S3). This approach assumes that SNPs with larger importance scores have a greater chance of becoming real biomarkers than those with smaller scores. Through our perception of the biological processes, we noticed that GO enrichment analysis, using the top 50% of SNPs (374 for rectal bleeding and 183 for erectile dysfunction) for both endpoints, found more plausible biological processes. With the top 50% of SNPs, we repeated our model building process. There was no great performance improvement from 0.70 to 0.71 AUC for rectal bleeding, whereas for erectile dysfunction there was relatively significant performance improvement from 0.62 to 0.65 AUC (Supplementary Figure S3). Alternatively, despite the increase in biological relevance, the improvement resulting from filtering out SNPs that are ranked lowly could also be viewed as a straightforward machine learning improvement step.

Figures 3C and D show directly connected protein-protein interaction networks for rectal bleeding and erectile dysfunction constructed using each list of nearby genes for the top 50% of SNPs. There are several mouse model studies that showed that Vitamin D receptor (VDR) deficiency is highly associated with rectal bleeding. Froicu *et al*. reported that VDR/IL10 knockout mice had severe rectal bleeding^35^ and similarly, Kong *et al*. reported that VDR deficiency resulted in an increased risk of mucosal damage and inflammatory bowel disease^36^. Their studies suggest an essential role of VDR in maintaining the intestinal mucosal barrier. There are several reports that showed a significant role of protein kinase C (PKC) and SMAD, shown in Fig. 3D, in erectile dysfunction. PKC is a key protein in regulating the contraction of vascular smooth muscle^37^,^38^. Wingard *et al*. showed in their animal model that PKC and Rho-kinase enhance vasoconstriction of the penile smooth muscle and contribute to erectile dysfunction^39^. Zhang *et al*. reported that overexpression of TGF-β1 and activation of the Smad signaling pathway in the penis of diabetic rats play a central role in deteriorating erectile dysfunction^40^.

As shown in Table 2, most biological processes implicated in rectal bleeding are involved in the ion transport activity. The intestinal epithelium regulates the absorption and secretion of salt and water via the activity of ion transporters^41^. Dysfunction of epithelial ion transport can be caused by a variety of factors such as inflammation and toxins, resulting in diarrheal illnesses. McCole *et al*. reported that ion transport is abnormal by decreased sodium absorption in mice with colitis and epidermal growth factor receptor (EGFR) activation can alleviate diarrhea associated with colitis^42^. Since rectal bleeding often accompanies diarrhea, the biological processes shown in Table 2 seem plausible and associated with rectal bleeding.

It is generally accepted that risk factors for erectile dysfunction and cardiovascular disease are similar, including age, smoking, high blood pressure, high cholesterol, and diabetes^43^. In our univariate analysis on the training data, age was significantly correlated with erectile dysfunction with p-value = 1.30 × 10^−5^, whereas smoking, diabetes, and hypertension were not statistically significant with p-values of 0.378, 0.410, and 0.522, respectively. ADT was also a significant predictor of erectile function as shown in many published articles. Through 19 articles found via PubMed (http://www.ncbi.nlm.nih.gov/pubmed/) literature searches, White *et al*. confirmed that RT/ADT can significantly worsen erectile dysfunction^44^. When we added age and ADT in the model of erectile dysfunction, the performance was further improved with 0.68 AUC. The analysis conducted by Banks *et al*. provided general support that erectile dysfunction could serve as a sentinel symptom for an increased risk of cardiovascular disease^45^. Vascular endothelium, as a main link between erectile dysfunction and cardiovascular disease, is critically involved in the regulation of blood circulation^46^. Therefore, dysfunction of the vascular endothelium may cause blood to inadequately flow to the erectile tissues, which eventually leads to erectile dysfunction. Dozio *et al*. reported that myeloperoxidase (MPO), a heme-containing enzyme that is released by neutrophils may contribute to endothelial dysfunction, thus leading to erectile dysfunction^47^. Penile erection is a hemodynamic process that is regulated by a balance between corporal smooth muscle relaxation and contraction^48^,^49^,^50^. Thus inability of this mechanism can be a cause of erectile dysfunction. Based on the studies mentioned above, it is plausible that the biological processes shown in Table 3 would be implicated in erectile dysfunction. The studies we cited were searched by a PubMed search engine with keywords, including the gene/protein names in Fig. 3C and D, biological process names in Tables 2 and 3, endpoint names (rectal bleeding and erectile dysfunction) or important clinical variables.

## Additional Information

**How to cite this article**: Oh, J. H. *et al*. Computational methods using genome-wide association studies to predict radiotherapy complications and to identify correlative molecular processes. *Sci. Rep.* **7**, 43381; doi: 10.1038/srep43381 (2017).

**Publisher's note:** Springer Nature remains neutral with regard to jurisdictional claims in published maps and institutional affiliations.

## Supplementary Material

Supporting Information

## Figures and Tables

**Table 1:** Number of samples used in the training and validation datasets in the model building process of rectal bleeding and erectile dysfunction.

Endpoint	Group	Training samples	Validation samples
Rectal bleeding	Cases	49	25
	Controls	194	97
	Total	243	122
Erectile dysfunction	Cases	88	45
	Controls	69	34
	Total	157	79

**Table 2:** Top 10 biological processes and corresponding genes for rectal bleeding.

Ranking	GO Processes/Genes	FDR
1	Regulation of ion transport	4.70E-06
	CACNA1D,CCL13,DPP10,DPP6,GCK,GNB4,GPR63,HOMER1,IL1RAPL1,JDP2,KCNIP4,KCNJ6,NLGN1,NOS1AP,PRKCB,PRKG1,VDR	
2	Regulation of potassium ion transport	5.33E-06
	CACNA1D,DPP10,DPP6,GCK,JDP2,KCNIP4,NOS1AP,PRKG1	
3	Regulation of metal ion transport	8.92E-06
	CACNA1D,CCL13,DPP10,DPP6,GCK,GNB4,GPR63,HOMER1,JDP2,KCNIP4,NOS1AP,PRKCB,PRKG1,VDR	
4	Regulation of cation transmembrane transport	1.76E-05
	CACNA1D,DPP10,DPP6,GNB4,HOMER1,KCNIP4,NOS1AP,PRKCB,PRKG1	
5	Regulation of potassium ion transmembrane transport	1.89E-05
	CACNA1D,DPP10,DPP6,KCNIP4,NOS1AP,PRKG1	
6	Regulation of ion transmembrane transport	2.31E-05
	CACNA1D,CCL13,DPP10,DPP6,GNB4,HOMER1,IL1RAPL1,KCNIP4,KCNJ6,NLGN1,NOS1AP,PRKCB,PRKG1	
7	Regulation of transmembrane transport	5.04E-05
	CACNA1D,CCL13,DPP10,DPP6,GNB4,HOMER1,IL1RAPL1,KCNIP4,KCNJ6,NLGN1,NOS1AP,PRKCB,PRKG1	
8	Cellular calcium ion homeostasis	1.27E-04
	CACNA1D,CCL1,CCL13,GCK,GNB4,GPR63,HERPUD1,PRKCB,PRKG1,TMEM165,VDR	
9	Regulation of system process	1.27E-04
	CACNA1D,CTNNA2,FST,GPR63,GUCY1A2,NLGN1,NOS1AP,PRKCB,PRKG1,SLC1A1,TENM4,TNFRSF21,TNR	
10	Regulation of ion transmembrane transporter activity	1.27E-04
	CACNA1D,GNB4,HOMER1,NLGN1,NOS1AP,PRKCB,PRKG1	

**Table 3:** Top 10 biological processes and corresponding genes for erectile dysfunction.

Ranking	GO Processes/Genes	FDR
1	Negative regulation of heart contraction	8.38E-10
	CXCR5,PDE4D,PRKCA,SPX	
2	Negative regulation of blood circulation	2.18E-08
	CXCR5,PDE4D,PRKCA,SPX	
3	Neutrophil chemotaxis	5.03E-08
	CXCR5,PDE4D,PRKCA	
4	Neutrophil migration	5.88E-08
	CXCR5,PDE4D,PRKCA	
5	Granulocyte chemotaxis	9.68E-08
	CXCR5,PDE4D,PRKCA	
6	Granulocyte migration	1.30E-07
	CXCR5,PDE4D,PRKCA	
7	Positive regulation of locomotion	2.63E-07
	CXCR5,DAB2IP,MAP2K1,OPRK1,PDE4D,PRKCA,SEMA5A,SMAD3	
8	Regulation of muscle system process	5.51E-07
	CXCR5,GLRX3,MAP2K1,PDE4D,PRKCA	
9	Regulation of muscle contraction	5.51E-07
	CXCR5,MAP2K1,PDE4D,PRKCA	
10	Positive regulation of cell migration	8.96E-07
	CXCR5,DAB2IP,MAP2K1,PDE4D,PRKCA,SEMA5A,SMAD3	
