# Supplementary material for: Computational methods using genome-wide association studies to predict radiotherapy complications and to identify correlative molecular processes
Source: Sci Rep. 2017 Feb 24;7:43381. doi: 10.1038/srep43381 (PMC5324069; doi:10.1038/srep43381)
Supplement: Supporting Information [file srep43381-s1.pdf]

Supplementary Information

Computational methods using genome-wide association studies to predict radiotherapy complications and to identify correlative molecular processes

Jung Hun Oh, Sarah Kerns, Harry Ostrer, Simon N. Powell, Barry Rosenstein & Joseph O. Deasy

Number of samples used in each tree construction

For an unbiased assessment of resulting predictive models, a dataset is split once and for all into two groups: a training dataset (2/3 of samples) and a validation dataset (1/3 of samples). For example, for 365 evaluable patients in an endpoint of rectal bleeding, 243 and 122 samples are used for training and validation, respectively. We employ 5-fold cross-validation (CV). At each iteration of CV, 4 folds (243×0.8≈194 samples) are used for modeling and the resulting model is tested using the hold-out validation data. In random forest regression, each tree is constructed using a bootstrapping dataset that is randomly sampled with replacement from the original data, having the same size as the original data. On average, 36.8% samples, called the out-of-bag (OOB) data, are excluded in the process of constructing each tree. Therefore, in each tree construction, the size of out-of-bag (OOB) data is 71 (≈194×0.368) that are used to measure the SNP importance and 123 (≈194×0.632) samples are used for the tree construction.

Use of the pre-conditioned outcomes in place of the binary outcomes in our modeling

Let X denote a list of the top ranked SNPs that resulted in the best AUC using $k-1$ folds of the training data on cross validation in the model building process of rectal bleeding. Let Y^b and Y^c denote the binary outcomes and pre-conditioned outcomes that are computed using SNPs in X, respectively. We conducted Spearman's correlation tests between each SNP in X and the following parameters: Y^b, Y^c, and the first principal component computed using SNPs in X. **Figure S1** is a scatter plot of the comparison of these three correlations, showing linear patterns for each other. In particular, the linear pattern between the correlation of X vs. pre-conditioned outcomes and the correlation of X vs. original outcomes supports our idea of using the pre-conditioned outcomes in the model building process, implying that it is likely that the residual indicates the denosing effect of the original outcomes.

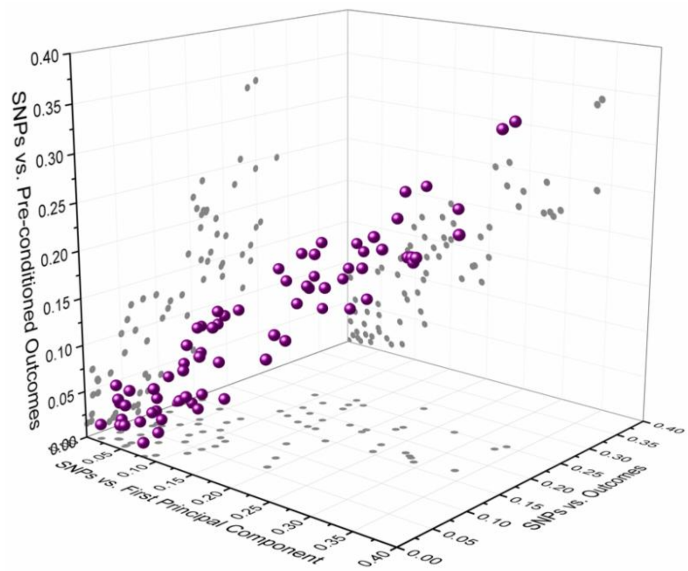

Figure S1. Comparison of the correlation between each SNP in X and the following parameters: 1) Y^{b}, 2) Y^{c}, and 3) the first principal component resulting from SNPs in X in the model building process of rectal bleeding where X indicates a list of the top ranked SNPs that resulted in the best AUC on the training data; Y^{b} indicates original outcomes; and Y^{c} indicates pre-conditioned outcomes. The value indicates the absolute value of Spearman's correlation coefficient.

Distribution of artifactual "noisy" SNPs

It is likely that a portion of SNPs selected in univariate analysis are false positive due to a relaxed p-value cutoff of 0.001. To investigate the distribution of potentially artifactual "noisy" SNPs for rectal bleeding, we generated 60,000 synthetic SNPs using the GWAsimulator (http://biostat.mc.vanderbilt.edu/wiki/Main/GWAsimulator) with HapMap CEU data. With a p-value cutoff of 0.001, 74 SNPs remained. We combined these SNPs with 749 SNPs that were originally chosen in univariate analysis for rectal bleeding and iterated the model building process with the combined 823 SNPs. **Figure S2A** shows the SNP importance score obtained from the model building process. There was no noisy SNP within the top 26 SNPs, suggesting that the highly ranked SNPs are more likely to be real biomarkers. On the other hand, a broad range of ranking of noisy SNPs implies the possibility of the presence of noisy SNPs among 749 SNPs. **Figure S2B** shows the cumulative rate of observed SNPs starting from the top one, i.e., the number of observed SNPs/(the number of noisy SNPs + the number of observed SNPs). The rate reached the bottom between the top 400 and 500 SNPs.

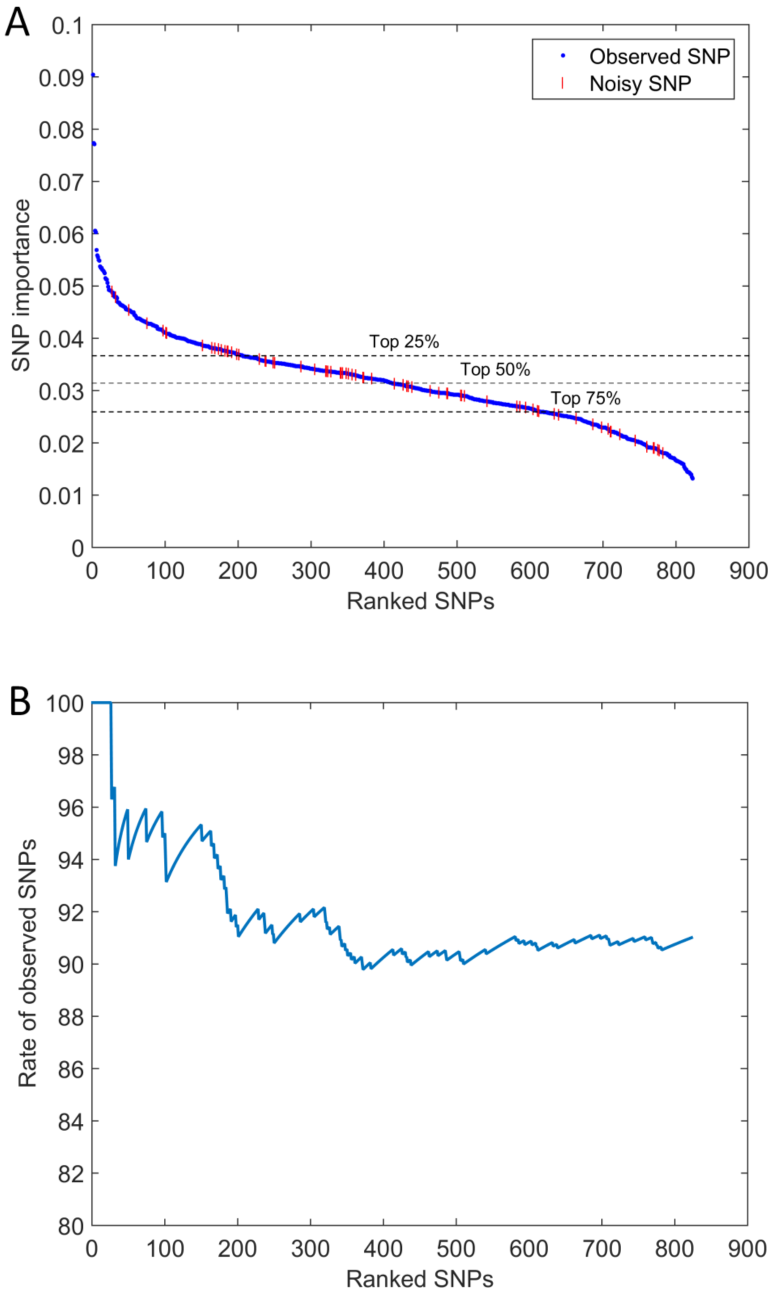

Figure S2. For rectal bleeding, (A) the SNP importance score and ranking when noisy SNPs were added in the model building process and (B) the rate of observed SNPs starting from the top SNP.

Comparison of the predictive power of models built using different set of SNPs

The model building process was iterated using the top {25%, 50%, 75%, and 100%} of SNPs according to the SNP importance score shown in **Figure 3**. The below figure shows the performance of predictive models on the hold-out validation data. We chose predictive models constructed using the top 50% of SNPs for both rectal bleeding and erectile dysfunction as final predictive models. Note that we chose the cutoff (50%) by investigating the biological relevance of biological processes to the endpoints, not by the predictive performance. The predictive power was slightly improved from 0.70 to 0.71 AUC for rectal bleeding, whereas there was relatively considerable improvement from 0.62 to 0.65 AUC for erectile dysfunction.

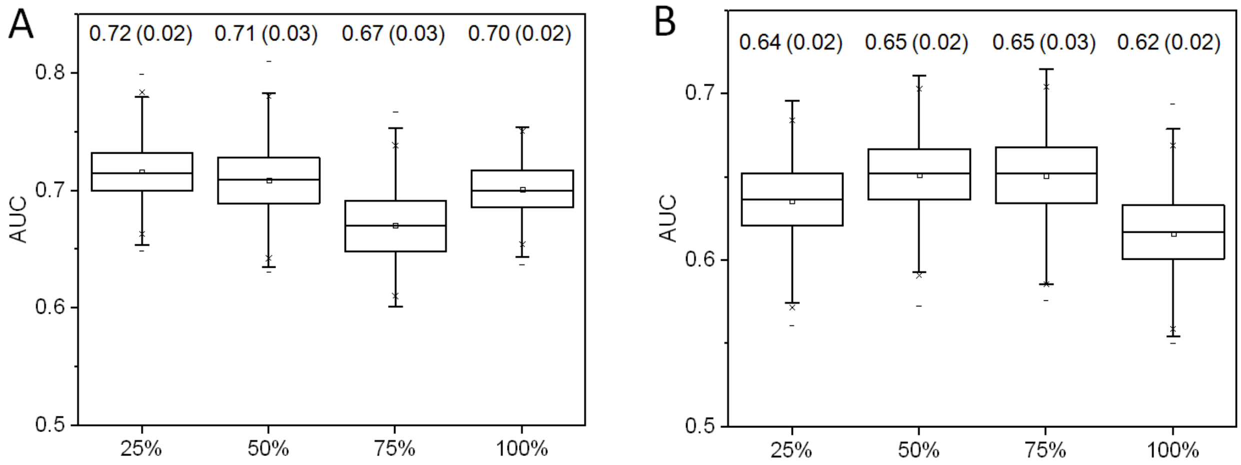

Figure S3. Box plots for the predictive power of the pre-conditioning random forest regression on the hold-out validation data in (A) rectal bleeding and (B) erectile dysfunction when the top {25%, 50%, 75%, and 100%} of SNPs according to the SNP importance score measure in the model building process were used.

Finding a set of optimal SNPs based on biological relevance analysis

We identified nearby genes for the top {25%, 50%, 75%, and 100%} of SNPs according to the SNP importance score shown in **Figure 3.** With these genes, we performed gene ontology enrichment analysis to identify biological processes using the MetaCore software. After perceptual analysis for these biological processes, we decided that those biological processes that are enriched by the genes identified from the top 50% of SNPs are more relevant to both endpoints. For example, *muscle tissue development* found using the top 25% of SNPs is less likely to be associated with rectal bleeding.

Likewise, *regulation of heart contraction* and *regulation of blood circulation* found using the top 75% and 100% of SNPs do not appear to be associated with rectal bleeding. However, genes identified by the top 50% of SNPs seem to act in biological processes more relevant to rectal bleeding. For erectile dysfunction, *learning or memory* found using the top 75% and 100% of SNPs is less likely to be associated with erectile dysfunction. In contrast, several biological processes including *negative regulation of heart contraction*, *negative regulation of blood circulation*, and *regulation of muscle contraction*, found using the top 50% of SNPs, appear to be more associated with erectile dysfunction than those found using the top 25% of SNPs.

Assessment of population structure effects

The following table shows the number of patients with and without toxicities across populations. A chi-square test was performed to investigate whether there is a significant difference in the number of events (toxicities) between populations. As a result, there was no significant difference between populations for both endpoints with p-value = 0.37 and 0.43 for rectal bleeding and erectile dysfunction, respectively.

Endpoint	Toxicity	African - American	Hispanic	Caucasian	Asian	Unknown
Rectal bleeding	No	42	28	209	3	9
	Yes	12	8	50	3	1
Erectile dysfunction	No	9	8	81	1	4
	Yes	18	16	96	1	2

Algorithm S1. The proposed multi-SNP predictive model algorithm.

1. Split a dataset into 2/3 and 1/3 of samples for training and validation, respectively

2. Using the training dataset, perform k-fold cross validation (CV)

Using $k - 1$ folds whose binary outcomes are y^b, do univariate analysis using logistic regression and calculate AUC^U for each of the m SNPs as follows:

2.1 With an increasing number of ranked SNPs based on AUC^U, perform principal component analysis (PCA)

2.1.1 Using 1 or 2 principal components, perform logistic regression fitted to y^b

2.1.2 Denote the predicted outcomes as y^c and calculate AUC^M for y^b and y^c

2.1.3 Exit this loop when AUC^M reaches a predefined threshold

2.2 Find the y^{c*} that produces the best AUC^M

2.3 Build a pre-conditioned random forest regression (PRFR) model with the y^{c*} and m SNPs

2.4 Test the resulting predictive model using the hold-out validation dataset whose binary outcomes are y^{bV} and calculate AUC^V for PRFR's predicted outcomes (y^{cV}) and y^{bV}

3. To estimate performance variance, repeat steps 2.1-2.4 and tabulate variability of AUCs on the hold-out validation dataset

---

AUC^U: AUC in univariate analysis using logistic regression on a given k -fold CV partition

AUC^M: AUC in logistic regression analysis with the first or first few principal components

AUC^V: AUC calculated on the hold-out validation dataset

y^b: Original binary outcomes (toxicity vs. non-toxicity) for samples belonging to $k - 1$ folds on CV

y^{bV}: Original binary outcomes for the hold-out validation dataset

y^c: Pre-conditioned continuous outcomes predicted by logistic regression with the first or first few principal components

y^{c*}: Pre-conditioned continuous outcomes that result in the best AUC^M

y^{cV}: Final PRFR's predicted outcomes for the hold-out validation dataset

Table S1. Top 10 biological processes identified using each list of genes that were found by the top {25%, 50%, 75%, and 100%} of SNPs according to the SNP importance score measured in the model building process for rectal bleeding.

Top 25% of SNPs

Ranking	GO Processes	FDR
1	second-messenger-mediated signaling	1.033E-03
2	muscle tissue development	1.138E-03
3	positive regulation of calcium ion transport	5.122E-03
4	system development	1.325E-02
5	striated muscle tissue development	1.752E-02
6	synapse organization	1.752E-02
7	cGMP biosynthetic process	1.752E-02
8	protein localization to synapse	1.752E-02
9	membrane repolarization during SA node cell action potential	1.752E-02
10	phospholipase C-activating G-protein coupled glutamate receptor signaling pathway	1.752E-02

Top 50% of SNPs

Ranking	GO Processes	FDR
1	regulation of ion transport	4.70E-06
2	regulation of potassium ion transport	5.33E-06
3	regulation of metal ion transport	8.92E-06
4	regulation of cation transmembrane transport	1.76E-05
5	regulation of potassium ion transmembrane transport	1.89E-05
6	regulation of ion transmembrane transport	2.31E-05
7	regulation of transmembrane transport	5.04E-05
8	cellular calcium ion homeostasis	1.27E-04
9	regulation of system process	1.27E-04
10	regulation of ion transmembrane transporter activity	1.27E-04

Top 75% of SNPs

Ranking	GO Processes	FDR
1	regulation of system process	9.845E-08
2	regulation of ion transport	3.671E-07
3	regulation of heart contraction	6.915E-07
4	regulation of blood circulation	6.915E-07
5	regulation of cation transmembrane transport	1.320E-06
6	regulation of ion transmembrane transport	1.320E-06
7	second-messenger-mediated signaling	1.815E-06
8	regulation of transmembrane transport	3.167E-06
9	regulation of metal ion transport	4.303E-06
10	regulation of muscle contraction	8.274E-06

Top 100% of SNPs

Ranking	GO Processes	FDR
1	regulation of ion transport	8.887E-10
2	regulation of system process	7.061E-09
3	regulation of ion transmembrane transport	1.040E-08
4	regulation of transmembrane transport	2.715E-08
5	second-messenger-mediated signaling	2.715E-08
6	regulation of heart contraction	4.412E-08
7	regulation of blood circulation	4.756E-08
8	regulation of cation transmembrane transport	9.550E-08
9	regulation of metal ion transport	1.072E-07
10	regulation of transporter activity	1.465E-07

Table S2. Top 10 biological processes identified using each list of genes that were found by the top {25%, 50%, 75%, and 100%} of SNPs according to the SNP importance score measured in the model building process for erectile dysfunction.

Top 25% of SNPs

Ranking	GO Processes	FDR
1	locomotion	2.191E-06
2	cell projection morphogenesis	2.191E-06
3	neuron development	2.191E-06
4	neuron differentiation	2.191E-06
5	blood vessel development	2.191E-06
6	cell part morphogenesis	2.191E-06
7	regulation of endothelial cell migration	2.191E-06
8	movement of cell or subcellular component	2.702E-06
9	vasculature development	2.889E-06
10	anatomical structure formation involved in morphogenesis	2.896E-06

Top 50% of SNPs

Ranking	GO Processes	FDR
1	negative regulation of heart contraction	8.376E-10
2	negative regulation of blood circulation	2.180E-08
3	neutrophil chemotaxis	5.026E-08
4	neutrophil migration	5.883E-08
5	granulocyte chemotaxis	9.684E-08

6	granulocyte migration	1.300E-07
7	positive regulation of locomotion	2.631E-07
8	regulation of muscle system process	5.510E-07
9	regulation of muscle contraction	5.510E-07
10	positive regulation of cell migration	8.960E-07

Top 75% of SNPs

Ranking	GO Processes	FDR
1	negative regulation of heart contraction	5.553E-09
2	negative regulation of blood circulation	1.423E-07
3	neutrophil chemotaxis	2.536E-07
4	neuron differentiation	2.536E-07
5	generation of neurons	2.536E-07
6	neutrophil migration	2.536E-07
7	granulocyte chemotaxis	4.452E-07
8	learning or memory	5.003E-07
9	neurogenesis	5.003E-07
10	granulocyte migration	5.003E-07

Top 100% of SNPs

Ranking	GO Processes	FDR
1	activation of phospholipase C activity	5.100E-09
2	learning or memory	5.100E-09
3	negative regulation of heart contraction	5.100E-09
4	positive regulation of phospholipase C activity	8.574E-09
5	regulation of phospholipase C activity	1.148E-08
6	cognition	1.151E-08
7	single-organism behavior	1.338E-08
8	positive regulation of phospholipase activity	7.723E-08
9	neuron differentiation	7.769E-08
10	negative regulation of blood circulation	7.769E-08

Table S3. SNP ranking that was sorted based on the SNP importance score measured in the model building process for rectal bleeding. SNPs were assigned to a gene if they are located within 10kb upstream and downstream of the gene.

Ranking	SNP	Nearby genes	Importance score
1	rs2825233		0.097
2	rs333167	NFIA	0.082
3	rs7008185	RGS22	0.080

4	rs10227348		0.065
5	rs2404149	HOMER1	0.065
6	rs2591101		0.065
7	rs10519411		0.064
8	rs1994701		0.063
9	rs12553697	SLC1A1, SPATA6L	0.063
10	rs747209		0.062
11	rs3850762	MICU3	0.061
12	rs2551214	MYT1L	0.061
13	rs154238	SEC13	0.059
14	rs13138652		0.058
15	rs13136965		0.058
16	rs1817124	AGBL1	0.058
17	rs12711386	TBC1D9	0.057
18	rs33967331	OXCT1	0.057
19	rs13167578	C5orf51	0.057
20	rs17062849		0.056
21	rs859357	TNR	0.056
22	rs12607893		0.056
23	rs6881873	OXCT1	0.056
24	rs3757572	TBRG4	0.056
25	rs6450138	FST	0.056
26	rs4725080		0.055
27	rs862242	ATP6AP1L	0.055
28	rs11877437	RIT2	0.055
29	rs2551215	MYT1L	0.055
30	rs862245	ATP6AP1L	0.054
31	rs13315469	TNIK	0.054
32	rs6894520	ATP6AP1L	0.053
33	rs13168035	OXCT1	0.053
34	rs1445587		0.053
35	rs10891664	REXO2	0.053
36	rs447881	CDH12	0.053
37	rs11126163		0.052
38	rs7950087		0.052
39	rs7181082		0.052
40	rs13104417	ADAM29	0.052
41	rs7658139	ADAM29	0.052
42	rs1343007	FRMD4A	0.052
43	rs11679340	SLC8A1	0.052
44	rs16871955	OXCT1	0.051
45	rs2285264	MICU3	0.051
46	rs5949642		0.051

47	rs7698088	TBC1D9	0.051
48	rs1864803		0.051
49	rs2949942	PITPNC1	0.050
50	rs2300584	GCK	0.050
51	rs12334614		0.050
52	rs17309228		0.050
53	rs7746208	TAB2	0.050
54	rs9408158		0.050
55	rs11155629	TAB2	0.050
56	rs17529367		0.049
57	rs2206006	CHM	0.049
58	rs734512		0.049
59	rs1944390		0.049
60	rs2219207	NXPE1	0.049
61	rs6072084	MAFB	0.049
62	rs8013023	JDP2	0.049
63	rs7281316		0.049
64	rs8103535	DPRX	0.049
65	rs9866601		0.049
66	rs11750607	OXCT1, C5orf51	0.048
67	rs2822832		0.048
68	rs10990245		0.048
69	rs1995413		0.048
70	rs16923500	UBXN2B, CYP7A1	0.048
71	rs16905107		0.048
72	rs16871972	OXCT1	0.048
73	rs35672	SEC13, GHRL	0.047
74	rs3123390	ZBTB18	0.047
75	rs10941556	OXCT1	0.047
76	rs6992117	TOX	0.047
77	rs444876	NTM	0.047
78	rs11209979		0.047
79	rs294559	MCTP2	0.047
80	rs4700799	ADAMTS2	0.047
81	rs7322542	TNFRSF19	0.047
82	rs12188447	CDH12	0.047
83	rs9926328		0.047
84	rs2328271		0.047
85	rs2278082	CCDC93	0.046
86	rs472587	DLG2	0.046
87	rs154237	SEC13	0.046
88	rs2729609		0.046
89	rs197003	IL1RAPL1	0.046

90	rs6725474	CCDC93	0.046
91	rs2061143	NXPE1	0.046
92	rs6683030		0.046
93	rs11082602		0.046
94	rs16867320		0.046
95	rs264174	PIEZO2	0.045
96	rs1028867	PALM2	0.045
97	rs10519410		0.045
98	rs17458235		0.045
99	rs7615625	TNIK	0.045
100	rs2639313		0.045
101	rs7649134	PDZRN3	0.045
102	rs268706	TARS	0.045
103	rs3787255	EYA2	0.045
104	rs4713926	ADTRP	0.045
105	rs9465923	CDKAL1	0.045
106	rs10894446	NTM	0.045
107	rs8108694	ZNF543, ZNF304	0.045
108	rs11211945	GUCY1A2	0.045
109	rs9885072	CDH12	0.044
110	rs9790641	RBM47	0.044
111	rs680944	SH3BGR	0.044
112	rs7228876		0.044
113	rs3808900	LHX6, RBM18	0.044
114	rs4650390		0.044
115	rs2768275		0.044
116	rs1357395		0.044
117	rs11876485		0.044
118	rs1186048	CCDC171	0.044
119	rs9487199	GPR63	0.044
120	rs1576672		0.044
121	rs12333397		0.044
122	rs1510744		0.044
123	rs9635948		0.044
124	rs12150846		0.044
125	rs11590310	NTNG1	0.044
126	rs516888	SH3BGR	0.043
127	rs4794281	CA10	0.043
128	rs2457846	FRMD4A	0.043
129	rs741038	GCK	0.043
130	rs41330144		0.043
131	rs2653648		0.043
132	rs715345		0.043

133	rs979220	CACNA1D	0.043
134	rs2812510	C10orf35	0.043
135	rs4073251		0.043
136	rs1061615	PDPN	0.043
137	rs17087870		0.043
138	rs2852949		0.043
139	rs1593054	NDUFS4, FST	0.043
140	rs16976240		0.043
141	rs1153744	PPP1R1C	0.043
142	rs4928149	FILIP1L	0.043
143	rs10833229	NAV2	0.043
144	rs1459920		0.043
145	rs10483303		0.043
146	rs934882		0.042
147	rs430924	CDH12	0.042
148	rs9828340		0.042
149	rs10023965	GABRB1	0.042
150	rs12670403	AHR	0.042
151	rs17471738	SGIP1	0.042
152	rs2889548	ADCY8	0.042
153	rs6795317	SCHIP1	0.042
154	rs3862777	DLG2	0.042
155	rs12439884		0.042
156	rs1773015		0.042
157	rs9571314		0.042
158	rs2562126	HERPUD1	0.042
159	rs17782181	DOCK9	0.042
160	rs1999468		0.042
161	rs2714656		0.042
162	rs12482280		0.042
163	rs6807031	MITF	0.041
164	rs10505560	ADCY8	0.041
165	rs10512542		0.041
166	rs4808203	GATAD2A	0.041
167	rs3875341		0.041
168	rs10102682		0.041
169	rs9613021	MYO18B	0.041
170	rs17053449	SGCD	0.041
171	rs6413472	CTH	0.041
172	rs2870749		0.041
173	rs6671294		0.041
174	rs12425885	BTBD11	0.041
175	rs13354232		0.041

176	rs2449447		0.041
177	rs7226130	SPAG9	0.041
178	rs934207		0.041
179	rs284732		0.041
180	rs2251301	MTMR9	0.041
181	rs2230600	PTPN13	0.041
182	rs12618287		0.040
183	rs550830	HRH4	0.040
184	rs16969506	MGAT5B	0.040
185	rs16860627		0.040
186	rs10891647	NNMT	0.040
187	rs715346		0.040
188	rs11981812	DPP6	0.040
189	rs7699581		0.040
190	rs10010361	GRID2, ATOH1	0.040
191	rs2616072	C10orf35	0.040
192	rs4653062		0.040
193	rs4402749		0.040
194	rs17080199	GFPT2	0.040
195	rs834771		0.040
196	rs715347		0.040
197	rs1008695	LRP1B	0.040
198	rs6787045	MAGI1	0.040
199	rs12353518		0.040
200	rs6424638		0.040
201	rs2916498	CTNNA2	0.040
202	rs6943978	SDK1	0.040
203	rs10809832		0.040
204	rs10470587		0.040
205	rs473005	TMEM165	0.040
206	rs7797162	ELMO1	0.040
207	rs7098507		0.039
208	rs6717960		0.039
209	rs2385833		0.039
210	rs2019991		0.039
211	rs9317478		0.039
212	rs17573253	CCT4	0.039
213	rs17049410		0.039
214	rs12278023	DLG2	0.039
215	rs6072196		0.039
216	rs159319	CCL13, CCL1	0.039
217	rs11899768		0.039
218	rs3793753	PIP4K2A	0.039

219	rs12150848		0.039
220	rs2714657		0.039
221	rs13337852		0.039
222	rs17078797		0.039
223	rs6480585	PRKG1	0.039
224	rs12452891	MGAT5B	0.039
225	rs12693239		0.039
226	rs515092	BLID	0.039
227	rs1941378	TENM4	0.039
228	rs16997454	TSHZ2	0.039
229	rs10914991		0.039
230	rs12449066	WWOX	0.039
231	rs11187371	MYOF	0.039
232	rs16860030		0.039
233	rs17552411	AHCYL2	0.039
234	rs7120482		0.039
235	rs3776763	STK10	0.039
236	rs2609836	FAM107B	0.039
237	rs17011727	CNTNAP5	0.039
238	rs532753	MGAT5B	0.039
239	rs631376	ALKBH8	0.038
240	rs6774615	SLC6A6	0.038
241	rs7944514	POLD3	0.038
242	rs11613346	TMEM132D	0.038
243	rs344404	CNTN4	0.038
244	rs10861733	BTBD11	0.038
245	rs2072070	CCL13, CCL1	0.038
246	rs159290	CCL1	0.038
247	rs7333447	SUGT1P3	0.038
248	rs13265608	CCDC26	0.038
249	rs159289	CCL1	0.038
250	rs13086738		0.038
251	rs12638862	TERC	0.038
252	rs4237397		0.038
253	rs2283380	RGS6	0.038
254	rs7612205	IGSF10	0.038
255	rs10505102	ANGPT1	0.038
256	rs6775136		0.038
257	rs6925604	REPS1	0.038
258	rs1449720		0.038
259	rs1490706		0.038
260	rs6553586		0.038
261	rs11713968		0.038

262	rs17142569		0.038
263	rs979438		0.038
264	rs4794194	SPAG9	0.038
265	rs13084396		0.038
266	rs4653061		0.038
267	rs13266795	CSMD1	0.038
268	rs2835862	KCNJ6	0.038
269	rs17142556		0.038
270	rs1806190		0.038
271	rs10959478		0.038
272	rs159313	CCL13	0.038
273	rs1863042	CCDC93	0.038
274	rs1825792	CDH12	0.037
275	rs7210332		0.037
276	rs155783	NDUFS4	0.037
277	rs1346376	SPOCK3	0.037
278	rs10419912	GATAD2A	0.037
279	rs10916762	VWA5B1	0.037
280	rs10157706	GADD45A	0.037
281	rs1805609		0.037
282	rs159291	CCL1	0.037
283	rs10485365		0.037
284	rs638196		0.037
285	rs11890841	DPP10	0.037
286	rs2087930		0.037
287	rs17555239		0.037
288	rs264536		0.037
289	rs2147384		0.037
290	rs3861854	KIAA0141	0.037
291	rs6072163		0.037
292	rs1958059	NPAS3	0.037
293	rs1033098		0.037
294	rs2279827	DRD3, ZNF80	0.037
295	rs17136316	ZNF75A	0.037
296	rs6587388	OR2T8, OR2W3	0.037
297	rs344651	LHFPL2	0.037
298	rs2122061		0.037
299	rs10010675	GRID2, ATOH1	0.036
300	rs4696975	KCNIP4	0.036
301	rs9812042		0.036
302	rs2853559	VDR	0.036
303	rs896313	DLG2	0.036
304	rs4727070	ZNF777	0.036

305	rs12196063		0.036
306	rs10873205	PLEKHH1	0.036
307	rs10255878		0.036
308	rs17719830	KIAA1468	0.036
309	rs2630182	FHIT	0.036
310	rs4689626	SORCS2	0.036
311	rs12546003		0.036
312	rs4982398	NDRG2	0.036
313	rs1186387	CCDC171	0.036
314	rs2094520	CCDC171	0.036
315	rs10796186		0.036
316	rs2178333	REPS1	0.036
317	rs1350836		0.036
318	rs4457339	TNKS	0.036
319	rs1341739	CCDC171	0.036
320	rs10794203		0.036
321	rs7485210	ADGRD1	0.036
322	rs275010		0.036
323	rs2303329	CCDC93	0.036
324	rs6677050	PTPN14	0.036
325	rs2548649	SNX18	0.036
326	rs10970529	RFX3	0.035
327	rs8089756		0.035
328	rs3729904	PRKCB	0.035
329	rs2975272	ZNF250, ZNF16	0.035
330	rs16976243		0.035
331	rs11593477		0.035
332	rs9567894		0.035
333	rs583173	NLGN1	0.035
334	rs4838988	MAGI3	0.035
335	rs10517596	LRAT	0.035
336	rs1169475	CCDC171	0.035
337	rs2332329		0.035
338	rs754574	GNB4	0.035
339	rs13390621	ANTXR1, GFPT1	0.035
340	rs10938039		0.035
341	rs12038219	CD247, CREG1	0.035
342	rs1184913	CCDC171	0.035
343	rs17668620	PTPRD	0.035
344	rs10800279	NOS1AP, OLFML2B	0.035
345	rs7653813		0.035
346	rs9473031	TNFRSF21	0.035

347	rs6799873		0.035
348	rs11123477		0.035
349	rs1868606		0.035
350	rs16977561	CGNL1	0.035
351	rs5747295	ATP6V1E1, BCL2L13	0.035
352	rs7917012		0.035
353	rs10030477		0.035
354	rs1169476	CCDC171	0.035
355	rs7199566	SNTB2	0.035
356	rs868031	CTNNA2	0.035
357	rs17041944		0.035
358	rs6800052	CNTN6	0.035
359	rs17101326	WDR89	0.035
360	rs1254729		0.035
361	rs4694846	GABRB1	0.035
362	rs17140083	RBFOX1	0.035
363	rs874869	FBXO11	0.035
364	rs1787318	MYO5B	0.035
365	rs582096	MGAT5B	0.035
366	rs153398	SREK1	0.035
367	rs11042789	AMPD3	0.035
368	rs6449150	CC2D2A	0.035
369	rs7013751	CDH17	0.035
370	rs133553		0.035
371	rs17298157		0.035
372	rs17142139		0.034
373	rs4964576	BTBD11	0.034
374	rs11876970		0.034
375	rs15047	PRMT2	0.034
376	rs896739		0.034
377	rs10773460		0.034
378	rs11187345		0.034
379	rs11082590		0.034
380	rs1118823	FGG, FGA	0.034
381	rs1787319	MYO5B	0.034
382	rs249621		0.034
383	rs6449973		0.034
384	rs3136681	CCL13, CCL1	0.034
385	rs12571538	RBM17, PFKFB3	0.034
386	rs10490128		0.034
387	rs7708775	PJA2	0.034
388	rs13393217	HDAC4	0.034

389	rs4744313	PTPDC1	0.034
390	rs3961050		0.034
391	rs3732477	CPN2	0.034
392	rs7619608	ECT2	0.034
393	rs11136920		0.034
394	rs6755898	SP110	0.034
395	rs11187341		0.034
396	rs6779873	CNTN6	0.034
397	rs1550353	GRID2, ATOH1	0.034
398	rs4791610	CDRT7	0.034
399	rs12637201	ECT2	0.034
400	rs4904509	FOXN3	0.033
401	rs9625253		0.033
402	rs6651056		0.033
403	rs10508824		0.033
404	rs17638180	AKAP13	0.033
405	rs2624917		0.033
406	rs1860164	UPP2	0.033
407	rs1393735	CA10	0.033
408	rs12465358	UPP2	0.033
409	rs12464424		0.033
410	rs6857138	GRID2, ATOH1	0.033
411	rs17841189	SH3GL3	0.033
412	rs1866069	TTC41P	0.033
413	rs7042626		0.033
414	rs6058224	UQCC1, EIF6	0.033
415	rs1293114		0.033
416	rs5943527	IL1RAPL1	0.033
417	rs7916658		0.033
418	rs355820		0.033
419	rs7067945		0.033
420	rs17691848		0.033
421	rs6501805	KIAA0195	0.033
422	rs12515663	PCDH1	0.033
423	rs17513709	NSUN7	0.033
424	rs7181796	AKAP13	0.033
425	rs12734251	CHD5	0.033
426	rs13180690	PDE4D	0.033
427	rs133204	LRP5L	0.033
428	rs10508823		0.032
429	rs2812509	C10orf35	0.032
430	rs10452607	TRERF1, MRPS10	0.032
431	rs2434781	CDH18	0.032

432	rs2692815	SFMBT2	0.032
433	rs9294979	THBS2	0.032
434	rs7139809		0.032
435	rs10497826		0.032
436	rs6943606		0.032
437	rs3741044	AMPD3	0.032
438	rs6632412	CFAP47	0.032
439	rs9806191	DAPK2	0.032
440	rs6971319	MKLN1	0.032
441	rs12918362		0.032
442	rs882733	NSUN7	0.032
443	rs7846356	MYOM2	0.032
444	rs4316801	CA10	0.032
445	rs2670199		0.032
446	rs5912422	FAM46D	0.032
447	rs7652461		0.032
448	rs17541282		0.032
449	rs12655617	PDE4D	0.032
450	rs16967577		0.032
451	rs9960012		0.032
452	rs4888901	WWOX	0.032
453	rs1582007		0.032
454	rs2096823		0.032
455	rs7903506	DLG5	0.032
456	rs1927060	CHMP1B2P	0.032
457	rs17082323	SYNE1	0.031
458	rs1680887		0.031
459	rs931950	BRE, FLJ31356	0.031
460	rs514253	CLUL1, CETN1	0.031
461	rs17461863	GABRB1	0.031
462	rs9576167	SUPT20H, CSNK1A1L	0.031
463	rs738633	RBX1	0.031
464	rs313438		0.031
465	rs3816432	YPEL2	0.031
466	rs2299262	PON1	0.031
467	rs7847020		0.031
468	rs6765863	ECT2, SPATA16	0.031
469	rs1379257	CEP70	0.031
470	rs6716246		0.031
471	rs11662963	KIAA1468	0.031
472	rs10816139	PTPRD	0.031
473	rs11102736	SYT6	0.031

474	rs1347064	CHMP1B2P	0.031
475	rs6933331	MAS1L, UBD	0.031
476	rs17059161		0.031
477	rs188025		0.031
478	rs2878682	CFAP47	0.031
479	rs7937598	TSPAN18	0.031
480	rs6113032		0.031
481	rs5913245	CHMP1B2P	0.031
482	rs423779	CACNA1E	0.031
483	rs9862461	RAB7A	0.031
484	rs1128272	GKN2	0.031
485	rs6854244		0.031
486	rs12215832		0.031
487	rs187615		0.031
488	rs1680885		0.031
489	rs7230449		0.031
490	rs12027327	SYT6	0.031
491	rs7553348	TNNI3K	0.030
492	rs12723898	TUFT1	0.030
493	rs9494089		0.030
494	rs13169888	UBTD2	0.030
495	rs16948801	WWOX	0.030
496	rs10816138	PTPRD	0.030
497	rs6482959	FOXI2	0.030
498	rs4978888	C9orf152	0.030
499	rs17034615	KIF1B	0.030
500	rs17006475	PLAC8, COPS4	0.030
501	rs9946520		0.030
502	rs543977	PIGN	0.030
503	rs1684900	CCDC6	0.030
504	rs10450540	CAMK1D	0.030
505	rs17247902		0.030
506	rs3095634	AKTIP	0.030
507	rs1778214		0.030
508	rs565610	PIGN	0.030
509	rs973877		0.030
510	rs2449448		0.030
511	rs9303606		0.030
512	rs870570		0.030
513	rs12199325		0.030
514	rs11783232		0.030
515	rs3103161	CDH12	0.030
516	rs2711078	PKP4	0.030

517	rs17509502		0.030
518	rs2333856	IPCEF1	0.030
519	rs17045259		0.030
520	rs17801966	AKTIP	0.030
521	rs1747649		0.030
522	rs7314936		0.030
523	rs7644832		0.029
524	rs10903923		0.029
525	rs6106629		0.029
526	rs241798		0.029
527	rs17289606		0.029
528	rs7296628	CMKLR1	0.029
529	rs261076	FAM196B	0.029
530	rs7436353	RXFP1	0.029
531	rs6756459		0.029
532	rs2065841	NFIA	0.029
533	rs12703626	TPK1	0.029
534	rs7206996	YPEL2	0.029
535	rs10896	MEF2BNB	0.029
536	rs2446830	CDH17	0.029
537	rs11251645		0.029
538	rs7377646	RXFP1	0.029
539	rs4825704	CUL4B, MCTS1	0.029
540	rs457062	DENND4C	0.029
541	rs7377670	RXFP1	0.029
542	rs11251481		0.029
543	rs17289585		0.029
544	rs428786		0.029
545	rs17130592	WLS	0.029
546	rs7253030		0.029
547	rs17512637	RBM47	0.029
548	rs1805502	GRIN2B	0.029
549	rs7634745	CEP70	0.029
550	rs2284428	GRIN2B	0.029
551	rs10836419	PAMR1	0.029
552	rs10219717	CCDC63	0.029
553	rs7824301		0.029
554	rs9987094	ZFAT	0.029
555	rs2179538	IPCEF1	0.028
556	rs4464774		0.028
557	rs11662257	LOXHD1	0.028
558	rs12099529		0.028
559	rs1383879		0.028

560	rs7830390	LRRCC1	0.028
561	rs10759078	PTPRD	0.028
562	rs6632414	CFAP47	0.028
563	rs16865763		0.028
564	rs7089611	KIAA1217	0.028
565	rs6629021	CFAP47	0.028
566	rs12582270	SOX5	0.028
567	rs9342869		0.028
568	rs10991000		0.028
569	rs17383845	SHC4	0.028
570	rs3801004	FSCN1	0.028
571	rs11578843		0.028
572	rs11251641		0.028
573	rs2859481	BATF	0.028
574	rs6709269	RAB11FIP5	0.028
575	rs17216862		0.028
576	rs10505625	ZFAT	0.028
577	rs2041102	OR7A5, OR7C1	0.028
578	rs17062816		0.028
579	rs2239253	RGS6	0.028
580	rs17023680	CNTN4	0.028
581	rs4665631		0.028
582	rs697702		0.027
583	rs900973	SLC14A2	0.027
584	rs10772506	ETV6	0.027
585	rs17107113	JAKMIP2	0.027
586	rs923074	SLC14A2	0.027
587	rs306536	DDX31	0.027
588	rs4629440		0.027
589	rs7519127	TTLL7	0.027
590	rs17563756		0.027
591	rs1965670	PJA2	0.027
592	rs1419644	MAS1L, UBD	0.027
593	rs17755728	TENM4	0.027
594	rs3790227	SLC24A3	0.027
595	rs10977800	PTPRD	0.027
596	rs17000271		0.027
597	rs11848155		0.027
598	rs7659958	MAN2B2	0.027
599	rs17054187		0.027
600	rs11176735		0.027
601	rs4502644		0.027
602	rs2546589		0.027

603	rs10903922		0.027
604	rs11174192	FAM19A2	0.026
605	rs12082808		0.026
606	rs10979437		0.026
607	rs2417923		0.026
608	rs7049635		0.026
609	rs2268129	GRIN2B	0.026
610	rs7705858	NADK2, RANBP3L	0.026
611	rs739854		0.026
612	rs11104497		0.026
613	rs1495008		0.026
614	rs12652757	ADRB2	0.026
615	rs7902402		0.026
616	rs2685257		0.026
617	rs9360487		0.026
618	rs5749006	CECR3	0.026
619	rs877387		0.026
620	rs10903925		0.026
621	rs955245		0.026
622	rs4869474	RANBP3L	0.026
623	rs12683129	HACD4, IFNB1	0.026
624	rs9930917		0.026
625	rs7105434		0.026
626	rs3772932	GAP43	0.026
627	rs7849713	HACD4, IFNB1	0.026
628	rs16909281	FAM135B	0.025
629	rs1931365	WNK2	0.025
630	rs5934426	VCX3B	0.025
631	rs10990940		0.025
632	rs10761206	WNK2	0.025
633	rs10889769	DEPDC1, RPE65	0.025
634	rs6021721	ZFP64	0.025
635	rs17013484		0.025
636	rs1924529		0.025
637	rs4809721	PREX1	0.025
638	rs2942135	PLEKHA6	0.025
639	rs1480053		0.025
640	rs7775829	PHACTR2	0.025
641	rs6824699		0.025
642	rs17104350	ESRRB	0.024
643	rs1045392	FAM173B	0.024
644	rs1438631	GAP43	0.024
645	rs7133438	NOS1	0.024

646	rs7788756		0.024
647	rs2575515		0.024
648	rs12107704		0.024
649	rs1952052		0.024
650	rs12549436		0.024
651	rs17484734	NALCN	0.024
652	rs10511277		0.024
653	rs11765212		0.024
654	rs17155665	CACNA2D1	0.023
655	rs5934151		0.023
656	rs7269050		0.023
657	rs8063167	RMI2	0.023
658	rs11859377	IRX3	0.023
659	rs10888857	ACOT11	0.023
660	rs7849485	HACD4, IFNB1	0.023
661	rs7163029	LINGO1	0.023
662	rs6744735		0.023
663	rs709188		0.023
664	rs11614312	MED13L	0.023
665	rs13097883	CCDC39	0.023
666	rs7329268		0.023
667	rs6640144	VCX3B	0.023
668	rs2046902	ASIC2	0.023
669	rs10899964		0.023
670	rs9537297		0.023
671	rs12084736	SKI	0.023
672	rs9838500	PDCD6IP	0.023
673	rs9932807	NAA60, C16orf90	0.022
674	rs5968699	CHM	0.022
675	rs11994169		0.022
676	rs16885752	C5orf67	0.022
677	rs6096776	ZFP64	0.022
678	rs8133540	RCAN1	0.022
679	rs12109225	WDR70, NUP155	0.022
680	rs1380813	KCNIP4	0.022
681	rs7158201	ADAM20, MED6	0.022
682	rs4732418	CACNA2D1	0.022
683	rs6815858		0.022
684	rs7134341	SRRM4	0.022
685	rs4772838		0.022
686	rs1585687	COL23A1	0.022
687	rs6535972		0.021
688	rs6497853		0.021

689	rs7521055	RSPO1, C1orf109	0.021
690	rs2834772		0.021
691	rs16967866	GRB2, KIAA0195	0.021
692	rs17729263		0.021
693	rs8090945	DCC	0.021
694	rs9576769		0.021
695	rs7983935		0.021
696	rs11166624	ZFAT	0.021
697	rs6927607		0.021
698	rs12451314	ASIC2	0.021
699	rs16944395		0.021
700	rs2628644		0.020
701	rs17771094	ZFAT	0.020
702	rs4710979		0.020
703	rs11794984		0.020
704	rs17770070	ZFAT	0.020
705	rs16987857	HS1BP3	0.020
706	rs11056813	SLC15A5	0.020
707	rs8095200		0.020
708	rs11897377	GCFC2	0.020
709	rs8052035		0.019
710	rs4762546	ANKS1B	0.019
711	rs2633674	POU1F1	0.019
712	rs12213204		0.019
713	rs9459317		0.019
714	rs6500685		0.019
715	rs7644156		0.019
716	rs11714171	ERC2	0.019
717	rs7609420	GCFC2	0.019
718	rs1919561		0.019
719	rs2047201	SPATA5	0.019
720	rs2036936		0.018
721	rs17081861		0.018
722	rs2741607	DBNDD2, TP53TG5	0.018
723	rs3860271		0.018
724	rs1434364		0.018
725	rs7181017	KLHL25	0.018
726	rs10937080		0.018
727	rs10955035	RAB11FIP1	0.018
728	rs4950128		0.018
729	rs17628762		0.018
730	rs224671		0.017
731	rs12712927	SIX3	0.017

732	rs11829822	TMPO, SLC25A3	0.017
733	rs11743007	ERGIC1	0.017
734	rs17074756		0.017
735	rs17629141		0.017
736	rs224666		0.017
737	rs10064691	TBCA, OTP	0.017
738	rs9296115	PACSIN1	0.016
739	rs7746427		0.016
740	rs11832465	SRRM4	0.016
741	rs1196348	SNX27	0.016
742	rs10488742		0.016
743	rs452755		0.016
744	rs6929303	SAYSD1	0.016
745	rs11578753		0.016
746	rs9356730		0.015
747	rs9358331		0.015
748	rs2037177	NEK11	0.015
749	rs2095268	EVI5	0.013

Table S4. SNP ranking that was sorted based on the SNP importance score measured in the model building process for erectile dysfunction. SNPs were assigned to a gene if they are located within 10kb upstream and downstream of the gene.

Ranking	SNP	Nearby genes	Importance score
1	rs319460	ATP8B1	0.139
2	rs463295	ATP8B1	0.135
3	rs17232178	GLRX3	0.131
4	rs4255740	MAP2K1	0.127
5	rs7149188		0.126
6	rs11017104	GLRX3	0.126
7	rs10829695	GLRX3	0.126
8	rs10764930	GLRX3	0.125
9	rs7082126	GLRX3	0.124
10	rs10829696	GLRX3	0.124
11	rs17775328		0.124
12	rs723187		0.124
13	rs6950259		0.124
14	rs7333648		0.123
15	rs4791505		0.114
16	rs7748820		0.113

17	rs317835	ATP8B1	0.111
18	rs1535904		0.111
19	rs301571		0.109
20	rs8080976	DNAH17	0.108
21	rs6846909		0.104
22	rs317838	ATP8B1	0.104
23	rs10113394		0.104
24	rs9525326	CHAMP1, UPF3A	0.103
25	rs1432441	MAP2K1	0.103
26	rs9678284		0.103
27	rs865332	ITK, FAM71B	0.103
28	rs7245988	NLRP11	0.102
29	rs4697682		0.102
30	rs4726411	DPP6	0.102
31	rs16949924	MAP2K1	0.102
32	rs2239865	MTMR7	0.101
33	rs815718		0.101
34	rs641461	COL8A1	0.100
35	rs598207	CXCR5	0.100
36	rs3760629	CLPTM1	0.100
37	rs4803781	CLPTM1	0.100
38	rs319461	ATP8B1	0.098
39	rs16949879	MAP2K1	0.097
40	rs4984390	MCTP2	0.097
41	rs8031897	TIPIN	0.097
42	rs7517337		0.096
43	rs11633291	MCTP2	0.096
44	rs11683069		0.095
45	rs4541064		0.094
46	rs1554931		0.094
47	rs755267		0.094
48	rs1344293		0.094
49	rs11694842	DNMT3A	0.093
50	rs183733	SEMA5A	0.093
51	rs756603	MTMR7	0.092
52	rs574079	DAB2IP	0.092
53	rs10237950		0.092
54	rs16959604	PRKCA	0.092
55	rs819238	ITK	0.092
56	rs9349145		0.092
57	rs12030578	USP48	0.091
58	rs1171594	MRLN	0.091

59	rs9822615		0.091
60	rs10472290		0.091
61	rs41007		0.091
62	rs7737213	ATP10B	0.090
63	rs514075	OPCML	0.090
64	rs12470828		0.090
65	rs11683506	SMARCAL1	0.090
66	rs1024969	ATP10B	0.090
67	rs4705493		0.089
68	rs6556502	ATP10B	0.089
69	rs6492521		0.089
70	rs2682966	FHIT	0.089
71	rs1022308	DLG2	0.089
72	rs8036023	MAP2K1	0.089
73	rs11869821	PRKCA	0.089
74	rs1750240		0.089
75	rs924506	ANKS1B	0.089
76	rs4921309	ATP10B	0.088
77	rs2836892		0.088
78	rs11871291	PRKCA	0.088
79	rs13430914		0.088
80	rs2075620	CLPTM1	0.087
81	rs4982676	SLC7A7	0.087
82	rs3093998	MICB	0.087
83	rs4354715	DLG2	0.087
84	rs2063972		0.087
85	rs2839241	PCNT	0.086
86	rs4712300	CAP2	0.086
87	rs9854419	CNTN4	0.086
88	rs9369181		0.086
89	rs261218		0.086
90	rs7292527		0.085
91	rs1554930		0.085
92	rs528526	DLEU7	0.085
93	rs11856789	CERS3	0.084
94	rs4346455		0.084
95	rs16930371	SSPN	0.084
96	rs6935608	PKHD1	0.084
97	rs17291801		0.084
98	rs11635009	CERS3	0.083
99	rs4235754		0.083
100	rs6678912		0.083
101	rs12550499		0.083

102	rs4624634		0.083
103	rs12537077	CUX1	0.082
104	rs10798052		0.082
105	rs6853616	TBC1D9	0.082
106	rs7766099		0.082
107	rs11952011	PDE4D	0.082
108	rs10979039		0.082
109	rs671549	DLEU7	0.082
110	rs10041627	CTNND2	0.082
111	rs1890473	EFCAB14	0.082
112	rs6885587	CTNND2	0.082
113	rs667046	EFCAB2	0.082
114	rs6880938	CTNND2	0.082
115	rs953018	SEMA5A	0.081
116	rs751853		0.081
117	rs12027256		0.081
118	rs12907997	SMAD3	0.081
119	rs1003271		0.081
120	rs9551134		0.081
121	rs1860994		0.081
122	rs11639209		0.081
123	rs2220746		0.080
124	rs4902923		0.080
125	rs10476200	TSPAN17	0.079
126	rs9408677	KANK1	0.079
127	rs10496216		0.079
128	rs7666487	GABRB1	0.079
129	rs10926895		0.079
130	rs2156973	FHIT	0.079
131	rs9364707		0.078
132	rs956211	CERS3	0.078
133	rs2605897		0.078
134	rs4681620	TSC22D2	0.078
135	rs6041590		0.078
136	rs3095341		0.078
137	rs10499676	ABCA13	0.078
138	rs917012	CTNND2	0.078
139	rs17278578		0.078
140	rs1440487	EFCAB14	0.078
141	rs611954		0.077
142	rs9553395		0.077
143	rs9553394		0.077
144	rs7988031		0.077

145	rs1922660		0.077
146	rs11670380		0.077
147	rs10749863	EFCAB14	0.077
148	rs7980124		0.077
149	rs33980476		0.076
150	rs2839258	PCNT	0.076
151	rs601060	TEX38	0.076
152	rs1012695		0.076
153	rs9380975		0.076
154	rs4673021	SGPP2	0.075
155	rs6114768	SYNDIG1	0.075
156	rs11148196		0.075
157	rs10947907		0.075
158	rs16918884	OPRK1	0.075
159	rs11721076	PDZRN3	0.074
160	rs11156961		0.074
161	rs9837013		0.074
162	rs4508584	SGPP2	0.073
163	rs12621723		0.073
164	rs5910076		0.073
165	rs6049771	SYNDIG1	0.073
166	rs5945702		0.073
167	rs6935166	ARHGAP18	0.073
168	rs2237182	ATXN1	0.073
169	rs10897895		0.073
170	rs665478	EFCAB2	0.073
171	rs11933507	FRAS1	0.073
172	rs7824784		0.072
173	rs2900724	THG1L	0.072
174	rs6859323	THG1L	0.072
175	rs9829886	FHIT	0.072
176	rs5629	PTGIS	0.072
177	rs894910		0.072
178	rs10505638		0.072
179	rs17482340	MYOF	0.072
180	rs721992	CCDC6	0.072
181	rs6078121		0.072
182	rs208867		0.072
183	rs10950076		0.071
184	rs7620877		0.071
185	rs1533896		0.071
186	rs1877796	CCDC6	0.071
187	rs4557574		0.071

188	rs10073056	CTNND2	0.070
189	rs1935474	PCDH15	0.070
190	rs10520849		0.070
191	rs11738432	LSM11	0.070
192	rs6850841		0.069
193	rs896025		0.069
194	rs2073221		0.069
195	rs9935982		0.069
196	rs7025024		0.068
197	rs632676	EFCAB2	0.068
198	rs7830977		0.068
199	rs7913010	MYOF, CEP55	0.068
200	rs6596147		0.068
201	rs10504070	C8orf22	0.068
202	rs10947863		0.068
203	rs1426535		0.068
204	rs2225692		0.068
205	rs4959710	MYLK4	0.067
206	rs16860083	GABRB1	0.067
207	rs6970554		0.067
208	rs10749338		0.066
209	rs9373088	SGK1	0.066
210	rs1321168	FYN	0.066
211	rs680507	EFCAB2	0.066
212	rs9328123	MYLK4	0.066
213	rs1321169	FYN	0.066
214	rs17391661	MYOF	0.065
215	rs4959711	MYLK4	0.065
216	rs6919449	MYLK4	0.065
217	rs1999898		0.065
218	rs17771145	PRKCA	0.065
219	rs2586072		0.064
220	rs10151135	ATXN3	0.064
221	rs3913521		0.064
222	rs2163895		0.064
223	rs6856348		0.064
224	rs17737996		0.064
225	rs6577695		0.063
226	rs183805		0.063
227	rs764077		0.063
228	rs11696845	KCNK15	0.063
229	rs3106324	MAML3	0.063
230	rs17547120		0.063

231	rs17076170		0.063
232	rs11882682	ARHGEF18	0.063
233	rs12126074		0.063
234	rs7062726		0.063
235	rs12991495	DNMT3A	0.063
236	rs1478001		0.062
237	rs11022045	ASCL2	0.062
238	rs1450138		0.061
239	rs16984110		0.061
240	rs17576620		0.061
241	rs10432780		0.060
242	rs877688		0.060
243	rs17050442		0.060
244	rs7827724		0.059
245	rs3913523		0.059
246	rs6922692		0.059
247	rs3913520		0.059
248	rs16984115		0.059
249	rs5907426		0.059
250	rs2168546		0.059
251	rs2666483	KANK4	0.058
252	rs4743859		0.058
253	rs17348299		0.058
254	rs11674467		0.058
255	rs6922617		0.058
256	rs17712825		0.058
257	rs6907281		0.058
258	rs2728475		0.058
259	rs7084796	TLL2	0.058
260	rs12109911		0.058
261	rs5964318		0.057
262	rs741661		0.057
263	rs4082418		0.057
264	rs4347920		0.057
265	rs725834	CLYBL	0.057
266	rs11751940		0.056
267	rs10826386		0.056
268	rs2153368		0.056
269	rs11055460		0.056
270	rs17592556		0.056
271	rs3914623		0.055
272	rs505699	CRTAC1	0.055
273	rs2075774	ANGEL1	0.055

274	rs1872692		0.055
275	rs795678		0.055
276	rs12125740		0.055
277	rs7739977		0.054
278	rs8102377	ZNF331	0.054
279	rs10820913		0.054
280	rs1341937		0.054
281	rs12491489		0.054
282	rs2385127	C8orf22	0.054
283	rs7590760	DNMT3A	0.053
284	rs16954046		0.053
285	rs17712566		0.053
286	rs2585607		0.053
287	rs10770822	SPX	0.053
288	rs467797		0.053
289	rs4779131		0.053
290	rs16954047		0.053
291	rs6489341	DCP1B	0.053
292	rs11857724		0.053
293	rs1201888		0.053
294	rs1201886		0.053
295	rs12315726	ETV6	0.052
296	rs2238128	ETV6	0.052
297	rs10518015		0.052
298	rs1423576	IL31RA	0.051
299	rs685909		0.051
300	rs10830363	TRIM77	0.051
301	rs1868677	ADGRG1	0.051
302	rs10830291		0.051
303	rs12242465	CRTAC1	0.050
304	rs11018662		0.050
305	rs7074375		0.050
306	rs6543012	TBC1D8	0.050
307	rs12526130		0.049
308	rs11726512	PALLD	0.049
309	rs4142494		0.049
310	rs12366176	NOX4	0.049
311	rs2388105		0.049
312	rs13059806		0.049
313	rs12930508		0.049
314	rs6543013	TBC1D8	0.049
315	rs11178702		0.049
316	rs10830288		0.048

317	rs12444374		0.048
318	rs1036140	DGKB	0.048
319	rs11686210		0.048
320	rs765304		0.048
321	rs11055430		0.048
322	rs11847171	CPSF2	0.047
323	rs4890018	RNF213	0.047
324	rs2407053		0.047
325	rs2922350		0.047
326	rs10009101	C4orf19	0.047
327	rs4425129	ZNF806	0.047
328	rs6055916	PLCB1	0.047
329	rs11055431		0.047
330	rs17330228	KCNH7	0.047
331	rs9787725	PCDH15	0.047
332	rs10826385		0.046
333	rs11076365		0.046
334	rs3848264		0.046
335	rs3885635	MPP7	0.046
336	rs10830359	TRIM77	0.046
337	rs10830321		0.046
338	rs11018670		0.045
339	rs12146625	FOLH1B	0.045
340	rs16906883		0.045
341	rs2917533	NOX4	0.045
342	rs983263		0.045
343	rs11018751	TRIM77	0.044
344	rs291090	PIGR	0.044
345	rs4685330	RFTN1	0.044
346	rs1201893		0.044
347	rs3757536	CADPS2	0.043
348	rs2031309		0.043
349	rs13419011		0.042
350	rs11755040	GPLD1	0.041
351	rs7879159	MAP3K15	0.041
352	rs17762851	AUTS2	0.040
353	rs12142899		0.040
354	rs11007656	SVIL	0.039
355	rs13356161	ADCY2	0.038
356	rs322896		0.038
357	rs2180958		0.037
358	rs877069	ADCY2	0.037
359	rs17604435		0.036

360	rs921574	OAF	0.036
361	rs10508357		0.035
362	rs5957142		0.035
363	rs10912092		0.032
364	rs7555613		0.031
365	rs1777604	CTTNBP2NL	0.030
366	rs1484623		0.030
367	rs12012376	IL1RAPL1	0.025
